# Effects of Clinicopathological Characteristics on the Survival of Patients Treated with PD-1/PD-L1 Inhibitor Monotherapy or Combination Therapy for Advanced Cancer: A Systemic Review and Meta-Analysis

**DOI:** 10.1155/2020/5269787

**Published:** 2020-12-18

**Authors:** Yuhan Wei, Yongfu Li, Qi Du, Xinyi Peng, Jiangtao Jin, Hong Guo, Yongyan Li, Qin Li

**Affiliations:** ^1^Department of Oncology, Beijing Friendship Hospital, Capital Medical University, Beijing 100050, China; ^2^Department of Oncology, The Second Affiliated Hospital of Hainan Medical University, Haikou 570311, China; ^3^Department of Cardiology, Beijing Chaoyang Hospital, Capital Medical University, Beijing 100020, China; ^4^Department of Intervention Therapy, Zezhou People's Hospital, Jincheng 048026, China; ^5^Department of Surgery, Beijing Changping District Hospital of Traditional Beijing Medicine, Beijing 102200, China; ^6^Department of Oncology, Beijing Pinggu Hospital, Beijing 101200, China

## Abstract

**Background:**

PD-1/PD-L1 inhibitors have made unprecedented progress in the treatment of cancer.

**Methods:**

A systemic search was conducted for randomized controlled trials that compared PD-1/PD-L1 inhibitor monotherapy or combination therapy with nonimmunotherapy. Hazard ratios (HRs) of overall survival (OS) according to the sex, age, ECOG PS, smoking status, liver metastasis, PD-L1 expression, EGFR, and KRAS status of patients were analyzed.

**Results:**

Totally, 13 studies with monotherapy and 5 with combination regimens were included, and the pooled HRs of OS were 0.74 (*P* < 0.001) and 0.64 (*P* < 0.001), respectively. EGFR wild-type patients could benefit from immunotherapy monotherapy (HR, 0.77; *P* < 0.001) while those of the mutant type had no survival benefit (HR, 1.11; *P* = 0.54), and the difference was statistically significant (interaction, *P* = 0.005). KRAS wild-type patients had no survival benefit from monotherapy (HR, 0.89; *P* = 0.49). For combination therapy, both male and female derived benefits but female had a significantly reduced risk of death (HR, 0.45; *P* < 0.001) compared with male (HR, 0.73; *P* < 0.001; interaction, *P* = 0.004). Nonsmokers derived more survival benefits from combination therapy (HR, 0.29; *P* < 0.001) than smokers (HR, 0.63; *P* = 0.001; interaction, *P* = 0.02). No significant difference was found between age, ECOG PS, liver metastasis, PD-L1 expression, and OS of both PD-1/PD-L1 inhibitor monotherapy and combination therapy.

**Conclusions:**

Both PD-1/PD-L1 inhibitor monotherapy and combination therapy significantly prolonged the OS of patients with advanced malignant tumors. EGFR status for monotherapy and sex and smoking status for combination therapy were important predictors of survival benefits.

## 1. Introduction

Immune checkpoint inhibitor (ICI) therapy has revolutionized cancer treatment due to its durable clinical response. Programmed cell death 1 (PD-1) and programmed cell death 1 ligand 1 (PD-L1) inhibitors, which target the PD-1/PD-L1 axis and reverse the negative regulators of T cells, are most promising and have been applied to the treatment of several types of cancers [1–5]. Nonetheless, a large proportion of patients do not derive benefit from this approach [6–8], so it is important to identify predictable biomarkers to select patients with the greatest potential benefit from immunotherapy.

It has been demonstrated that sex, age, performance status (PS), smoking status, and many other factors have impact on the human immune system, and studies have shown that these factors might affect the efficacy of PD-1/PD-L1 inhibitors [9–12]. Combination immunotherapy involves complex interactions of the immune system [13, 14], so these factors may play different roles in affecting immunotherapy monotherapy and combination therapy. Therefore, it is of great significance to explore the relationship between clinical or molecular factors and the efficacy of PD-1/PD-L1 inhibitor monotherapy and combination therapy for patients with advanced malignant tumors.

However, at present, studies exploring the interaction between clinicopathological features of patients and the efficacy of PD-1/PD-L1 inhibitor monotherapy and combination therapy separately are scarce. Here, we used the cumulative evidence of multiple clinical trials and conducted a meta-analysis to systematically assess the effect of these characteristics including sex, age, ECOG PS, smoking status, liver metastasis status, PD-L1 expression, epidermal growth factor receptor (EGFR), and Kirsten RAS (KRAS) status on overall survival (OS) of PD-1/PD-L1 inhibitor monotherapy and combination therapy for patients with advanced solid tumors.

## 2. Materials and Methods

### 2.1. Search Strategy

The study was conducted following the Preferred Reporting Items for Systematic Reviews and Meta-Analyses (PRISMA) guidelines [15]. A systemic search on PubMed, MEDLINE, and Embase from inception to September 30, 2019, was conducted for randomized controlled trials (RCTs) that compared PD-1/PD-L1 inhibitors with non-ICI or placebo. Two investigators (Y.H.W. and Q.D.) searched the database independently. The keywords included the following: (1) nivolumab, Opdivo, ONO-4538, MDX1106, BMS-936558; (2) pembrolizumab, lambrolizumab, Keytruda, MK-3475; (3) atezolizumab, Tecentriq, MPDL3280A; (4) durvalumab, Imfinzi, MEDI4736; (5) avelumab, Bavencio, MSB0010718C; and (6) checkpoint inhibitor, PD-1, PD-L1. The search was limited to a randomized controlled trial. We also searched the abstracts from the conference proceedings of the American Society of Clinical Oncology, the European Society for Medical Oncology, and the World Conference on Lung Cancer.

### 2.2. Selection Criteria

Exclusion and inclusion criteria were predetermined. Randomized control trials meeting the following criteria were eligible: (1) population: patients with unresectable stage III or IV solid tumors; (2) intervention: PD-1/PD-L1 inhibitor (pembrolizumab, nivolumab, atezolizumab, durvalumab, and avelumab) monotherapy or combination therapy; (3) control group: traditional therapy or placebo; and (4) outcome: hazard ratio (HR) of OS according to patient subgroups. We excluded single-arm phase I trials to avoid excessive heterogeneity. Only papers published in English were considered. When several articles of the same clinical trial appeared, only the last and/or the most complete reports were included. Two investigators (Y.H.W. and Y.F.L.) reviewed the retrieved list of articles independently. Differences were resolved through discussion and consensus with all researchers.

### 2.3. Data Extraction

All data was obtained from published manuscripts using a standardized data collection form. Two researchers (Y.H.W. and F.Y.L.) extracted data from the studies individually. Discrepancies were solved by discussion and consensus with all researchers. For each study, we extracted the study name, first author, year of publication, trial phase, tumor type, line of treatment, interventions of each group, patient number, and hazard ratio (HR) followed by 95% confidence interval (CI) of intention-to-treatment population and population in each subgroup analysis according to clinicopathological characteristics. The characteristics of interest for the study were sex, age, ECOG PS, smoking status, liver metastasis, PD-L1 expression (bounded by 1%), EGFR, and KRAS status.

The Cochrane risk of bias tool was used to evaluate the risk of bias for all included studies. Two authors evaluated the quality independently, and differences were settled through consultation.

### 2.4. Statistical Analysis

The analysis was performed using ReviewManager version 5.3 (the Nordic Cochrane Centre) by an inverse-variance-weighted method. Subgroup analysis was conducted to explore the effect of each factor. An interaction test was used to assess the difference in treatment efficacy within these subgroups.

Cochrane's *Q* statistic was used to evaluate the heterogeneity between studies, and *I*^2^ statistics were calculated. All reported *P* values were two-sided. *P* < 0.05 was considered statistically significant. The effect model was selected according to heterogeneity. The fixed effects model was applied when *P* ≥ 0.1 and *I*^2^ ≤ 50%; otherwise, the random effects model was applied.

## 3. Results

### 3.1. Search Results

**Figure 1:**
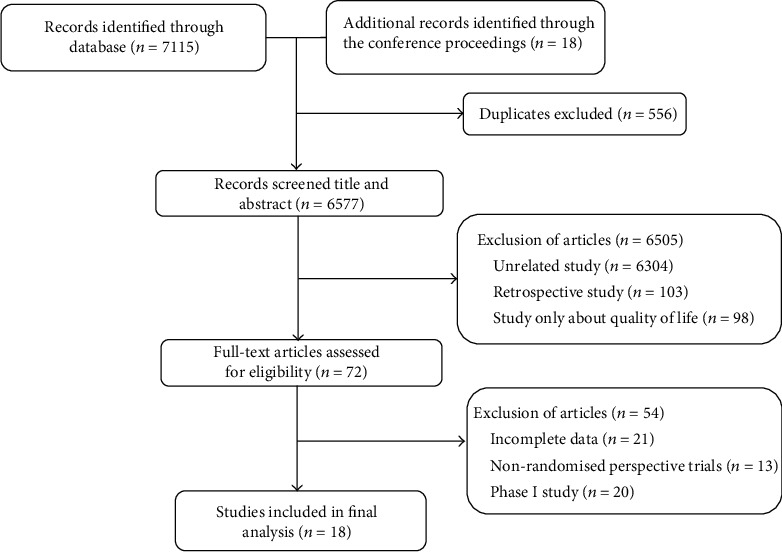
Flowchart diagram of the selection process for the trials.

Totally, 7133 relevant papers were collected during the preliminary search strategy. After review of the abstracts and full texts, 18 randomized controlled trials were included in the final analysis [1–3, 16–30] (Figure 1).

### 3.2. Characteristics of the Studies

The analysis included 18 studies, all of which were published within the last five years. We found 13 randomized controlled trials with PD-1/PD-L1 inhibitor monotherapy and 5 with combination regimens. There were 13 RCTs with PD-1 inhibitors (atezolizumab, 8; pembrolizumab, 5) and 5 with PD-L1 inhibitors (atezolizumab, 4; durvalumab, 1). Nine trials were conducted in patients with non-small-cell lung cancer, two each in renal cell carcinoma, urothelial cancer, and gastric cancer, and one each in patients with melanoma, squamous cell carcinoma of the head and neck, and small-cell lung cancer. There were a phase II trial,16 phase III trials, and a phase II/III trial. All RCTs enrolled patients with cancers of advanced or metastatic stage. Six were first-line treatments, and twelve were second-line or later. The number of patients for each eligible trial ranged from 287 to 1096. The main features of those trials are shown in Table 1. The result of quality evaluation of the included articles is shown in Table S1 in Supplementary Materials.

### 3.3. PD-1/PD-L1 Inhibitors on OS

Overall, the study consists of 10664 patients, 5870 (55%) of whom were included in the experimental group and 4794 (45%) were in the control group. Patients treated with PD-1/PD-L1 inhibitors were significantly associated with 29% reduction in the risk of death compared to the control group (HR, 0.71; 95% CI, 0.68-0.75; *P* < 0.001). In a monotherapy study of 7526 patients, 4080 received PD-1/PD-L1 inhibitors and 3446 were in the control group, and the pooled HR was 0.74 (95% CI, 0.69-0.78; *P* < 0.001) (Figure S1A in Supplementary Materials). In the combination study of 3138 patients, 1790 received immunotherapy and 1348 were in the control group. The pooled HR was 0.64 (95% CI, 0.57-0.71; *P* < 0.001) (Figure S1B in Supplementary Materials). We conducted survival analysis by tumor types as well, resulting in significantly prolonged OS in patients with non-small-cell lung cancer, renal cell carcinoma, urothelial cancer, gastric cancer, melanoma, squamous cell carcinoma of the head and neck, and small-cell lung cancer (pooled HRs were 0.69, 0.69, 0.80, 0.72, 0.42, 0.70 ,and 0.70, respectively) (Figure S2 in Supplementary Materials).

**Figure 2:**
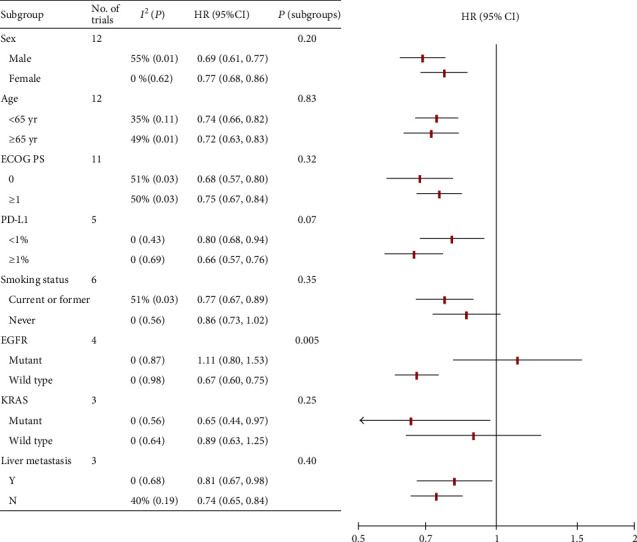
Subgroup analysis for monotherapy. HR: hazard ratio; Y: yes; N: no.

**Figure 3:**
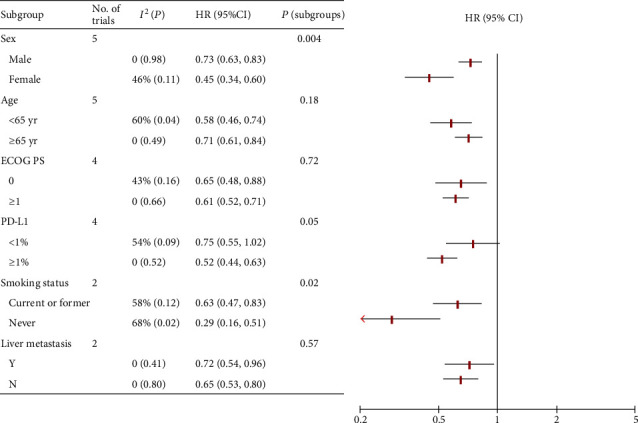
Subgroup analysis for combination therapy. HR: hazard ratio; Y: yes; N: no.

To thoroughly explore the effects of baseline on the survival of cancer patient treated with PD-1/PD-L1 inhibitors, further subgroup analysis for monotherapy and combination therapy were conducted separately and the results were shown in below part (summarized in Figures 2 and 3).

### 3.4. Subgroup Analysis for Monotherapy

**Figure 4:**
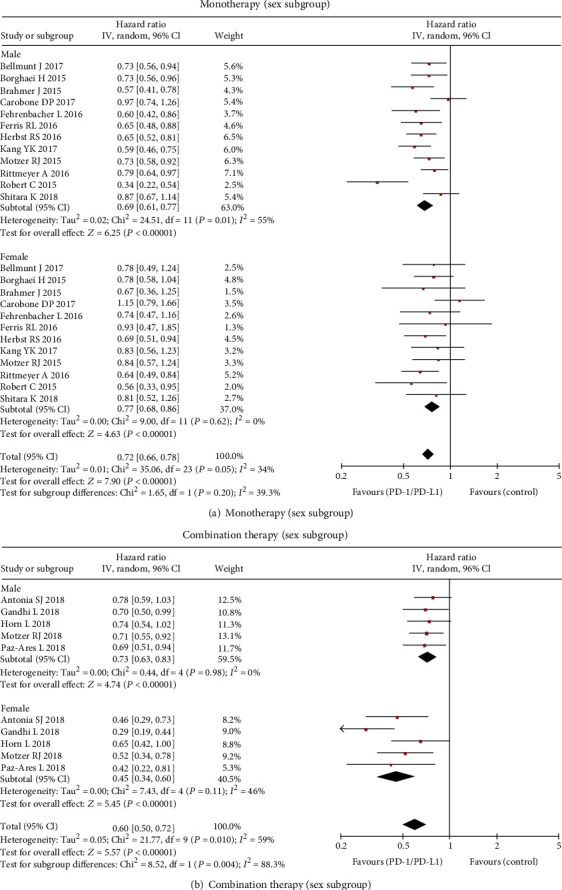
Forest plot of the hazard ratio comparing overall survival in patients who received PD-1/PD-L1 inhibitors versus control by sex: (a) monotherapy group and (b) combination therapy group.

The effect of sex on the efficacy of PD-1/PD-L1 inhibitor monotherapy was assessed in 12 RCTs, in which 4383 patients (66%) were males and 2212 (34%) were females. Studies showed that OS benefit of monotherapy was observed in both males (HR, 0.69; 95% CI, 0.61-0.77; *P* < 0.001) and females (HR, 0.77; 95% CI, 0.68-0.86; *P* < 0.001). There was no significant efficacy-sex interaction (*P* = 0.20) (Figures 2 and 4(a)).

There were 12 RCTs that analyzed the effect of age on the efficacy. The pooled HRs of OS were 0.74 (95% CI, 0.68-0.80; *P* < 0.001) in patients younger than 65 and 0.72 (95% CI, 0.66-0.79; *P* < 0.001) in patients not less than age 65 (interaction, *P* = 0.69) (Figure 2; Figure S3A in Supplementary Materials).

There were 11 RCTs reporting OS data according to ECOG PS. The PS = 0 group was composed of 2264 (37%) patients, and PS ≥ 1 was composed of 3892 (63%) patients. The pooled HR of OS for patients with ECOG PS = 0 was 0.68 (95% CI, 0.57-0.80; *P* < 0.001). The pooled HR of OS for patients with ECOG PS ≥ 1 was 0.75 (95% CI, 0.67-0.84; *P* < 0.001). There was no significant difference in survival between the two groups (*P* = 0.20) (Figure 2; Figure S4A in Supplementary Materials).

Six studies reported OS data for the smoking status subgroup. There were 2875 (77%) smokers and 838 (23%) nonsmokers, respectively. For the smoker group, the pooled HR was 0.77 (95% CI, 0.67-0.89; *P* < 0.001). For the nonsmoker group, the pooled HR was 0.86 (95% CI, 0.73-1.02; *P* = 0.08). There was no significant difference in survival between the two groups (*P* = 0.35) (Figure 2; Figure S5A in Supplementary Materials).

Three RCTs were included in the liver metastasis subgroup. For patients with liver metastasis (430 [22%]), the pooled HR was 0.81 (95% CI, 0.67-0.98; *P* = 0.03). For patients without liver metastasis (1535 [78%]), the pooled HR was 0.74 (95% CI, 0.65-0.84; *P* <0.001). No significant difference was observed between the two groups (interaction, *P* = 0.40) (Figure 2; Figure S6A in Supplementary Materials).

Five RCTs evaluated the effects of PD-L1 expression with 1% as the cut-off value. There were 987 (46%) and 1156 (54%) patients with PD-L1 expression less than 1% and not less than 1%, respectively. For the PD‐L1 < 1% group, the pooled HR was 0.80 (95% CI, 0.68-0.94; *P* = 0.005). For the PD‐L1 ≥ 1% group, the pooled HR was 0.66 (95% CI, 0.57-0.76; *P* < 0.001). There was no significant difference of OS benefit across PD-L1 subgroups (*P* = 0.07) (Figure 2; Figure S7A in Supplementary Materials).

Four RCTs were included in the EGFR status subgroup. For patients with EGFR mutant (272 [12%]), the pooled HR was 1.11 (95% CI, 0.80-1.53; *P* = 0.54). For patients with EGFR wild type (1990 [88%]), the pooled HR was 0.77 (95% CI, 0.67-0.89; *P* < 0.001). There was a significant difference in the efficacy of PD-1/PD-L1 inhibitors between EGFR mutant and wild-type groups (interaction, *P* = 0.005) (Figure 2; Figure S8A in Supplementary Materials).

The KRAS status was reported in three RCTs. For the KRAS mutant subgroup (148 [29%]), the pooled HR was 0.65 (95% CI, 0.44-0.97; *P* = 0.03). For the KRAS wild-type group (371 [71%]), the pooled HR was 0.89 (95% CI, 0.63-1.25; *P* = 0.49). There was no significant difference in survival between the two groups (interaction, *P* = 0.25) (Figure 2; Figure S8B in Supplementary Materials).

### 3.5. Subgroup Analysis for Combination Therapy

Five RCTs reported OS of combination therapy for the sex subgroup, in which 2194 patients (70%) were males and 944 (30%) were females. Female patients had a significantly reduced risk of death (HR, 0.45; 95% CI, 0.34-0.60; *P* < 0.001) compared with male (HR, 0.73; 95% CI, 0.63-0.83; *P* < 0.001; interaction, *P* = 0.004) when treated with PD-1/PD-L1 inhibitors as combination therapy versus the control group (Figures 3 and 4(b)).

Five studies conducted subgroup analyses by age. For patients under 65 years old (1698 [54%]), the pooled HR was 0.58 (95% CI, 0.46-0.74; *P* < 0.001). For patients aged 65 or above (1440 [46%]), the pooled HR was 0.71 (95% CI, 0.61-0.84; *P* < 0.001). No significant difference was observed between the two groups (interaction, *P* = 0.18) (Figure 3; Figure S3B in Supplementary Materials).

Survival data according to ECOG PS was available in four studies. For patients with PS = 0 (917 [40%]), the pooled HR was 0.65 (95% CI, 0.48-0.88; *P* = 0.005). For patients with PS ≥ 1 (1368 [60%]), the pooled HR was 0.61 (95% CI, 0.52-0.71; *P* < 0.001). No significant difference was observed between the two groups (interaction, *P* = 0.72) (Figure 3; Figure S4B in Supplementary Materials).

There were two RCTs reporting OS data for the smoking status subgroup. Among them, most patients (1192 [90%]) were smokers and 137 (10%) were nonsmokers. For the smoker group, the pooled HR was 0.63 (95% CI, 0.47-0.83; *P* = 0.001). For the nonsmoker group, the pooled HR was 0.29 (95% CI, 0.16-0.51; *P* < 0.001). Statistically significant difference in OS advantage between the two groups was observed (interaction, *P* = 0.02) (Figure 3; Figure S5B in Supplementary Materials).

Two RCTs evaluated the effects of liver metastasis on survival. There were 326 (26%) and 924 (74%) patients with liver metastasis and without liver metastasis, respectively. For patients with liver metastasis, the pooled HR was 0.72 (95% CI, 0.54-0.96; *P* = 0.02). For patients without liver metastasis, the pooled HR was 0.65 (95% CI, 0.53-0.80; *P* < 0.001). There was no statistically significant difference between the two groups (*P* = 0.57) (Figure 3; Figure S6B in Supplementary Materials).

Survival data according to PD-L1 expression was available in four studies. There were 1094 (47%) and 1258 (53%) patients with PD‐L1 < 1% and PD‐L1 ≥ 1%, respectively. The pooled HRs of PD-L1-negative patients and PD-L1-positive patients were 0.75 (95% CI, 0.55-1.02; *P* = 0.07) and 0.52 (95% CI, 0.44-0.63; *P* < 0.001), respectively. There was a near-significant difference in survival between the two groups (*P* = 0.05) (Figure 3; Figure S7B in Supplementary Materials).

## 4. Discussion

We conducted a meta-analysis of 18 RCTs involving 10909 patients to investigate the efficacy of PD-1/PD-L1 inhibitors in patients with advanced malignant tumors in different clinicopathological characteristic subgroups. The study demonstrated that PD-1/PD-L1 inhibitor monotherapy and combination therapy reduced the risk of death by 26% and 36% compared with the control group, respectively. For monotherapy, patients with EGFR wild-type tumors derived survival benefits while those with EGFR mutant tumors had no survival advantage, and there was statistically significant interaction between the EGFR status and the efficacy. Patients with tumors of KRAS mutant type had OS advantage to immunotherapy monotherapy compared to the control group while that of KRAS wild type had no survival benefit. However, no statistically significant interaction between the KRAS status and treatment effect was demonstrated. PD-L1-positive patients derived more benefits from PD-1/PD-L1 inhibitor monotherapy than PD-L1-negative patients did when compared with the control group, but with no significant difference. Survival benefit was independent of sex, age, performance status, and liver metastasis. For combination regimens, females derived more benefits than males. Nonsmokers and PD-L1-positive patients benefited more as well. Age, performance status, and liver metastasis could not predict the benefit of this approach.

The study confirmed that the PD-1/PD-L1 inhibitor improved the overall survival for both male and female with malignant cancers but female significantly benefited more than male did for combination therapy versus control. Notably, female showed greater survival benefits than male in every single trial of the combination therapy included in the analysis. On the contrary, there was a greater but not statistically significant benefit in male than in female when treated with PD-1/PD-L1 inhibitor monotherapy compared with control. Our conclusions were inconsistent with previous meta-analyses, among one of which showed that men were associated with more benefits when treated with ICIs [31] while others showed no statistically significant association of patient sex with the efficacy of ICIs [32, 33]. The discrepancy may be due to more data added in our analysis, and the strategy that we analyzed them separately according to monotherapy and combination regimens might be the main reason. The results confirmed that sex could be used to predict the curative effect of PD-1/PD-L1 inhibitor combination therapy, but there was not enough evidence to recommend it as a predictive biomarker for patients with monotherapy since no statistical difference was demonstrated.

Male and female have different innate and adaptive immune responses [34]. The sex-based differences of the immune system are probably due to the complex interactions among genes (sex chromosomes, RNAs, and genetic polymorphisms), hormones (oestradiol, progesterone, and androgens), and the environment [34–36]. There was preclinical evidence demonstrating that sex was an important variable in immunotherapy responses through differential regulatory T cell function and PD-L1 signaling [9]. In addition, a previous study demonstrated sex-associated difference in mutation burden [37]. The effect of sex on the efficacy of the PD-1/PD-L1 inhibitor is complex. The potential mechanism for the difference in efficacy between male and female is still unclear, and more studies are needed.

Consistent with previous reports [38, 39], the study confirmed that the efficacy of PD-1/PD-L1 inhibitors in elderly patients was comparable to that in younger patients, regardless of monotherapy or combination therapy. Immunosenescence is a phenomenon of the decline of immune function with aging, which might be associated with the poor efficacy of immunotherapy. Nonetheless, the association between PD-1/PD-L1 inhibitors and age-related immune changes is complex. On the one hand, T cell-mediated immune function is weakened with age, which results from multiple factors such as thymic atrophy, reduction of naive T cells, reduction of both T cell function, and T cell antigen recognition diversity [40, 41]. On the other hand, PD-L1 expression and tumor mutation burden increase with aging [42]. What is more, degeneration of organ function with aging makes it hard for elderly patients to tolerate chemotherapy. Therefore, even though differences in immune function between young and old people have been well studied, the potential impact of aging on immunotherapy response remains unclear and deserves further exploration.

The study revealed that smokers derived survival benefit from PD-1/PD-L1 inhibitor monotherapy versus the control group while benefit in nonsmokers was only marginal. On the contrary, for combination therapy versus the control group, nonsmokers had significantly more survival benefits than smokers. It is demonstrated that smoking causes a greater burden of cancer mutations [43]. Studies have shown significant differences in mutation patterns and frequencies of KRAS and EGFR genes between smokers and never smokers with lung cancer [44]. All of these might account for the results of monotherapy, but it could not be well explained for the reversed results of combination therapy. However, caution should be taken in the results of the combination therapy by the smoking status subgroup due to the limited number of included trials (*N* = 2). Future research is required to confirm the results.

The study demonstrated that an OS advantage of PD-1/PD-L1 inhibitor monotherapy and combination therapy versus control was observed for both patients with liver metastasis and without liver metastasis. The results are encouraging. The liver is a immuno-tolerant organ with a well-established mechanism of immune regulation, and patients with liver metastatic cancer are generally considered to be exempt from immunotherapy [12, 45]. However, our study confirmed that immunotherapy was a better choice for patients with liver metastasis compared with other treatments.

PD-L1 expression is considered the best biomarker to predict the efficacy of ICI. However, the predictive value of PD-L1 expression is still controversial [46, 47]. Our study showed that patients with high PD-L1 expression (1% as the cut-off value) showed more benefits to PD-1/PD-L1 inhibitor monotherapy and combination therapy versus control, but there was no significant difference. More studies are needed to explore the most appropriate cut-off value for different drugs and different drug regimens. In addition, the expression of PD-L1 is affected by many factors, and there is no unified method to detect PD-L1 expression in different experiments [46]. Further research is necessary for the exploration of the predictive value of PD-L1 expression.

Due to the lack of relevant data, we only discussed the effect of EGFR and KRAS status on survival benefits for monotherapy. Consistent with the previous study [48], our analysis showed that EGFR wild-type and KRAS mutant patients could significantly benefit from PD-1/PD-L1 inhibitors while EGFR mutant and KRAS wild-type patients could not. Thus, PD-1/PD-L1 inhibitors were not recommended for EGFR mutant and KRAS wild-type patients.

Our results have several important clinical and research implications. It might contribute to the selection of the appropriate patients for PD-1/PD-L1 inhibitor monotherapy and combination therapy and facilitate the design of future clinical trials. Sex, smoking status, EGFR status, and KRAS status should be taken into consideration in future study. Despite several achievements, caution should be taken in interpreting these results, for the fact that the study has several potential limitations. Firstly, it was based on published results rather than individual patient-level data. Secondly, heterogeneity between studies could not be fully avoided because of complicated interactions among these characteristics. Moreover, toxicity is also a key factor in the selection of treatment regimens. Since reports of adverse events for each subgroup were not available, it was unable to explore the impact of each factor on the adverse effects of PD-1/PD-L1 inhibitors.

## 5. Conclusions

The study demonstrated that both ICI monotherapy and combination therapy significantly prolonged the overall survival of patients with advanced malignant tumors. For monotherapy, patients with EGFR mutation and KRAS wild type were associated with no survival benefit. For combination therapy, sex and smoking status were important predictors for survival benefit. Our results might contribute to optimal treatment decision and reasonable clinical trial design.

## Figures and Tables

**Table 1:** Main characteristics of the studies included in the meta-analysis.

Study	Interventions	Tumor	Line	Patients, no. (%)							
				Male	<65	ECOG ≥ 1	PD‐L1 ≥ 1%	Smoker	EGFR wild type	KRAS wild type	No liver metastasis
Bellmunt J 2017	Pembrolizumab vs. ICC	Urothelial carcinoma	>1	402 (74)	230 (42)	—	230 (42)	351 (65)	—	—	355 (66)
Borghaei H 2015	Nivolumab vs. docetaxel	NS-NSCLC	>1	319 (55)	339 (58)	402 (69)	—	458 (79)	340 (81)	123 (66)	—
Brahmer J 2015	Nivolumab vs. docetaxel	S-NSCLC	>1	208 (76)	152 (56)	206 (76)	119 (53)	—	—	—	—
Carbone DP 2017	Nivolumab vs. ICC	NSCLC	1	332 (61)	281 (52)	362 (67)	—	475 (89)	—	—	—
Fehrenbacher L 2016	Atezolizumab vs. docetaxel	NSCLC	>1	169 (59)	174 (61)	193 (67)	195 (68)	231 (80)	147 (89)	45 (62)	—
Ferris RL 2016	Nivolumab vs. standard therapy	HNSCC	>1	300 (83)	248 (69)	287 (80)	149 (57)	—	—	—	—
Herbst RS 2016	Pembrolizumab vs. docetaxel	NSCLC	>1	634 (61)	604 (58)	689 (66)	—	—	875 (91)	—	—
Kang YK 2016	Nivolumab vs. placebo	Gastric or GOJ cancer	>1	348 (71)	284 (58)	350 (71)	—	—	—	—	387 (78)
Motzer RJ 2015	Nivolumab vs. everolimus	RCC	>1	619 (75)	497 (61)	—	—	—	—	—	—
Powles T 2018	Atezolizumab vs. chemotherapy	Urothelial carcinoma	>1	—	—	506 (54)	—	666 (72)	—	—	793 (85)
Rittmeyer A 2016	Atezolizumab vs. docetaxel	NSCLC	>1	520 (61)	453 (53)	535 (63)	463 (54)	694 (82)	628 (88)	203 (24)	—
Robert C 2015	Nivolumab vs. dacarbazine	Melanoma	1	246 (59)	200 (48)	148 (35)	—	—	—	—	—
Shitara K 2018	Pembrolizumab vs. paclitaxel	Gastric or GOJ cancer	>1	286 (72)	232 (59)	214 (54)	—	—	—	—	—
Antonia SJ 2018	Durvalumab+chemoradiotherapy vs. chemoradiotherapy	NSCLC	>1	500 (70)	391 (55)	362 (51)	303 (67)	649 (91)	—	—	—
Gandhi L 2018	Pembrolizumab+platinum vs. platinum	NS-NSCLC	1	363 (59)	312 (51)	347 (57)	388 (67)	543 (88)	—	—	—
Horn L 2018	Atezolizumab+carboplatin and etoposide vs. carboplatin and etoposide	SCLC	1	261 (65)	217 (54)	263 (65)	—	—	—	—	254 (63)
Motzer RJ 2018	Nivolumab+ipilimumab vs. sunitinib	RCC	1	615 (73)	524 (62)	—	214 (28)	—	—	—	670 (79)
Paz-Ares L 2018	Pembrolizumab+ICC vs. ICC	S-NSCLC	1	455 (81)	254 (45)	396 (71)	353 (63)	—	—	—	—

ICC: investigator's choice chemotherapy; NSCLC: non-small-cell lung cancer; NS-NSCLC: nonsquamous non-small-cell lung cancer; S-NSCLC: squamous non-small-cell lung cancer; HNSCC: squamous-cell carcinoma of the head and neck; GOJ: gastrooesophageal junction; RCC: renal cell carcinoma.

## Data Availability

All data generated or analyzed during this study are included in this published article (and its supplementary materials).

## Supplementary Materials

Supplementary Materials Table S1: risk of bias. Figure S1: forest plot of the hazard ratio comparing overall survival in patients who received PD-1/PD-L1 inhibitors versus control for the monotherapy group and combination therapy group. Figure S2: forest plot of the hazard ratio comparing overall survival in patients who received PD-1/PD-L1 inhibitors versus control by tumor types. Figure S3: forest plot of the hazard ratio comparing overall survival in patients who received PD-1/PD-L1 inhibitors versus control by age for (A) the monotherapy group and (B) the combination therapy group. Figure S4: forest plot of the hazard ratio comparing overall survival in patients who received PD-1/PD-L1 inhibitors versus control by ECOG PS for (A) the monotherapy group and (B) the combination therapy group. Figure S5: forest plot of the hazard ratio comparing overall survival in patients who received PD-1/PD-L1 inhibitors versus control by smoking status. (A) Monotherapy group and (B) combination therapy group. Figure S6: forest plot of the hazard ratio comparing overall survival in patients who received PD-1/PD-L1 inhibitors versus control by liver metastasis for (A) the monotherapy group and (B) the combination therapy group. Figure S7: forest plot of the hazard ratio comparing overall survival in patients who received PD-1/PD-L1 inhibitors versus control by PD-L1 expression for (A) the monotherapy group and (B) the combination therapy group. Figure S8: forest plot of the hazard ratio comparing overall survival in patients who received PD-1/PD-L1 inhibitors versus control for the monotherapy group by (A) EGFR status and (B) KRAS status.
