# Supplementary material for: Effects of Clinicopathological Characteristics on the Survival of Patients Treated with PD-1/PD-L1 Inhibitor Monotherapy or Combination Therapy for Advanced Cancer: A Systemic Review and Meta-Analysis
Source: J Immunol Res. 2020 Dec 18;2020:5269787. doi: 10.1155/2020/5269787 (PMC7762667; doi:10.1155/2020/5269787)
Supplement: Supplementary Materials — Table S1: risk of bias. Figure S1: forest plot of the hazard ratio comparing overall survival in patients who received PD-1/PD-L1 inhibitors versus control for the monotherapy group and combination therapy group. Figure S2: forest plot of the hazard ratio comparing overall survival in patients who received PD-1/PD-L1 inhibitors versus control by tumor types. Figure S3: forest plot of the hazard ratio comparing overall survival in patients who received PD-1/PD-L1 inhibitors versus control by age for (A) the monotherapy group and (B) the combination therapy group. Figure S4: forest plot of the hazard ratio comparing overall survival in patients who received PD-1/PD-L1 inhibitors versus control by ECOG PS for (A) the monotherapy group and (B) the combination therapy group. Figure S5: forest plot of the hazard ratio comparing overall survival in patients who received PD-1/PD-L1 inhibitors versus control by smoking status. (A) Monotherapy group and (B) combination therapy group. Figure S6: forest plot of the hazard ratio comparing overall survival in patients who received PD-1/PD-L1 inhibitors versus control by liver metastasis for (A) the monotherapy group and (B) the combination therapy group. Figure S7: forest plot of the hazard ratio comparing overall survival in patients who received PD-1/PD-L1 inhibitors versus control by PD-L1 expression for (A) the monotherapy group and (B) the combination therapy group. Figure S8: forest plot of the hazard ratio comparing overall survival in patients who received PD-1/PD-L1 inhibitors versus control for the monotherapy group by (A) EGFR status and (B) KRAS status. [file 5269787.f1.pdf]

Supplementary Materials

Table S1 Risk of bias

Study	Radom sequence generation	Allocation concealment	Blinding of participants and personel	Blinding of outcome assessment	Incompletement outcome data	Selective reporting	Other bias
Antonia SJ 2018	+	+	+	+	+	+	+
Bellmunt J 2017	+	+	-	-	+	+	+
Borghaei H 2015	+	+	-	-	+	+	+
Brahmer J 2015	+	+	?	?	+	+	+
Carbone DP 2017	+	+	-	-	+	+	+
Fehrenbacher L 2016	+	+	-	-	+	+	+
Ferris RL 2016	+	+	-	-	+	+	+
Gandhi L 2018	+	+	+	+	+	+	+
Herbst RS 2016	+	+	-	-	+	+	+
Horn L 2018	+	+	+	?	+	+	+
Kang YK 2017	+	+	+	+	+	+	+
Motzer RJ 2015	+	+	-	?	+	+	+
Motzer RJ 2018	+	+	-	+	+	+	+

Paz-Ares L 2018	+	+	+	+	+	+	+
Powles T 2018	+	+	-	-	+	+	+
Rittmeyer A 2016	+	+	-	-	+	+	+
Robert C 2015	+	?	+	+	+	+	+
Shitara K 2018	+	+	+	-	+	+	+

+ Low risk of bias; ? unclear risk of bias; - high risk of bias

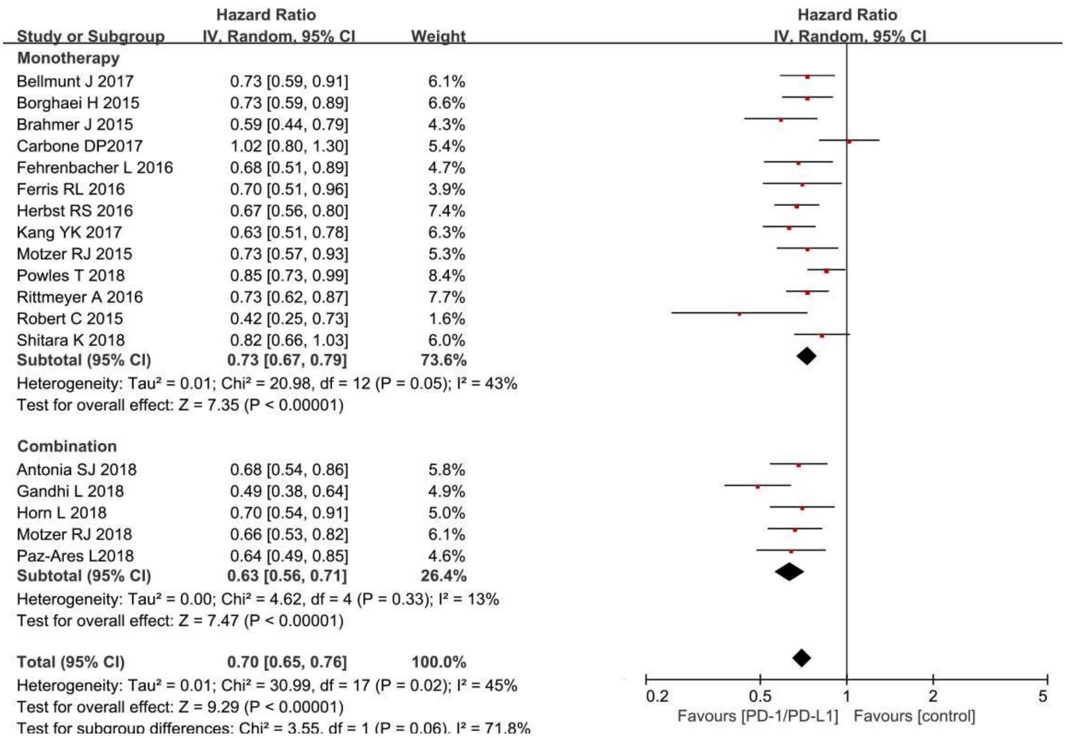

Figure S1 Forest plot of hazard ratio comparing overall survival in patients who received PD-1/PD-L1 inhibitors versus control for monotherapy group and combination therapy group.

Study or Subgroup	log[Hazard Ratio]	SE	Weight	Hazard Ratio IV, Random, 95% CI
**2.3.1 NSCLC**				
Antonia SJ 2018	-0.38566	0.1176	5.8%	0.68 [0.54, 0.86]
Borghaei H 2015	-0.3155	0.1042	6.6%	0.73 [0.59, 0.89]
Brahmer J 2015	-0.52763	0.1497	4.3%	0.59 [0.44, 0.79]
Carbone DP2017	0.019803	0.124	5.4%	1.02 [0.80, 1.30]
Fehrenbacher L 2016	-0.38744	0.141	4.7%	0.68 [0.51, 0.89]
Gandhi L 2018	-0.71335	0.1363	4.9%	0.49 [0.38, 0.64]
Herbst RS 2016	-0.40048	0.0915	7.4%	0.67 [0.56, 0.80]
Paz-Ares L2018	-0.44588	0.1416	4.6%	0.64 [0.49, 0.85]
Rittmeyer A 2016	-0.31471	0.087	7.7%	0.73 [0.62, 0.87]
**Subtotal (95% CI)**			**51.3%**	**0.69 [0.61, 0.77]**
Heterogeneity: Tau² = 0.02; Chi² = 18.43, df = 8 (P = 0.02); I² = 57%				
Test for overall effect: Z = 6.30 (P < 0.00001)				
**2.3.2 RCC**				
Motzer RJ 2015	-0.31471	0.1262	5.3%	0.73 [0.57, 0.93]
Motzer RJ 2018	-0.41552	0.1119	6.1%	0.66 [0.53, 0.82]
**Subtotal (95% CI)**			**11.4%**	**0.69 [0.59, 0.81]**
Heterogeneity: Tau² = 0.00; Chi² = 0.36, df = 1 (P = 0.55); I² = 0%				
Test for overall effect: Z = 4.43 (P < 0.00001)				
**2.3.3 UC**				
Bellmunt J 2017	-0.31471	0.1125	6.1%	0.73 [0.59, 0.91]
Powles T 2018	-0.16252	0.0778	8.4%	0.85 [0.73, 0.99]
**Subtotal (95% CI)**			**14.4%**	**0.80 [0.70, 0.93]**
Heterogeneity: Tau² = 0.00; Chi² = 1.24, df = 1 (P = 0.27); I² = 19%				
Test for overall effect: Z = 2.97 (P = 0.003)				
**2.3.4 GC**				
Kang YK 2017	-0.46204	0.1078	6.3%	0.63 [0.51, 0.78]
Shitara K 2018	-0.19845	0.1143	6.0%	0.82 [0.66, 1.03]
**Subtotal (95% CI)**			**12.3%**	**0.72 [0.55, 0.93]**
Heterogeneity: Tau² = 0.02; Chi² = 2.81, df = 1 (P = 0.09); I² = 64%				
Test for overall effect: Z = 2.53 (P = 0.01)				
**2.3.5 Melanoma**				
Robert C 2015	-0.8604	0.2779	1.6%	0.42 [0.25, 0.73]
**Subtotal (95% CI)**			**1.6%**	**0.42 [0.25, 0.73]**
Heterogeneity: Not applicable				
Test for overall effect: Z = 3.10 (P = 0.002)				
**2.3.6 HNSCC**				
Ferris RL 2016	-0.35667	0.1616	3.9%	0.70 [0.51, 0.96]
**Subtotal (95% CI)**			**3.9%**	**0.70 [0.51, 0.96]**
Heterogeneity: Not applicable				
Test for overall effect: Z = 2.21 (P = 0.03)				
**2.3.7 SCLC**				
Horn L 2018	-0.35667	0.1324	5.0%	0.70 [0.54, 0.91]
**Subtotal (95% CI)**			**5.0%**	**0.70 [0.54, 0.91]**
Heterogeneity: Not applicable				
Test for overall effect: Z = 2.69 (P = 0.007)				
**Total (95% CI)**			**100.0%**	**0.70 [0.65, 0.76]**
Heterogeneity: Tau² = 0.01; Chi² = 30.99, df = 17 (P = 0.02); I² = 45%				
Test for overall effect: Z = 9.29 (P < 0.00001)				
Test for subgroup differences: Chi² = 6.88, df = 6 (P = 0.33), I² = 12.8%				

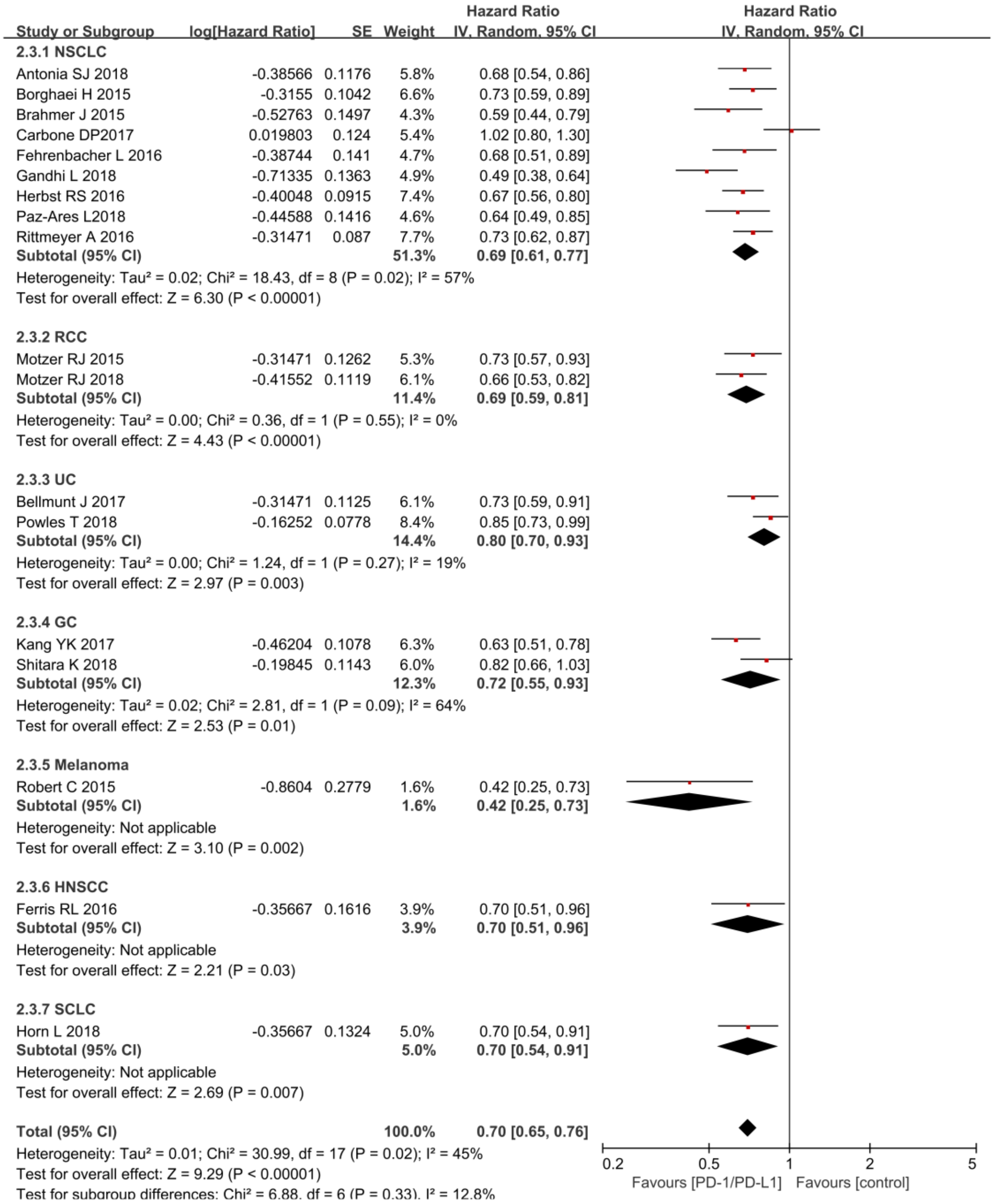

Figure S2 Forest plot of hazard ratio comparing overall survival in patients who received PD-1/PD-L1 inhibitors versus control by tumor types.

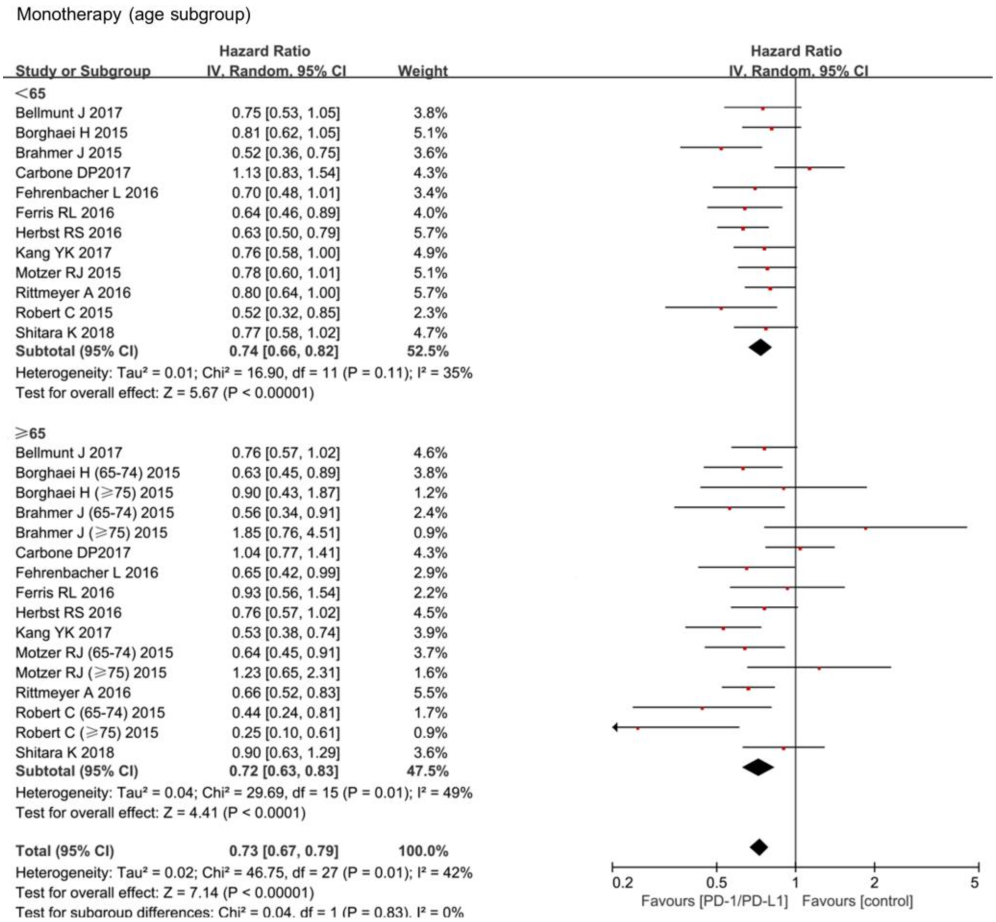

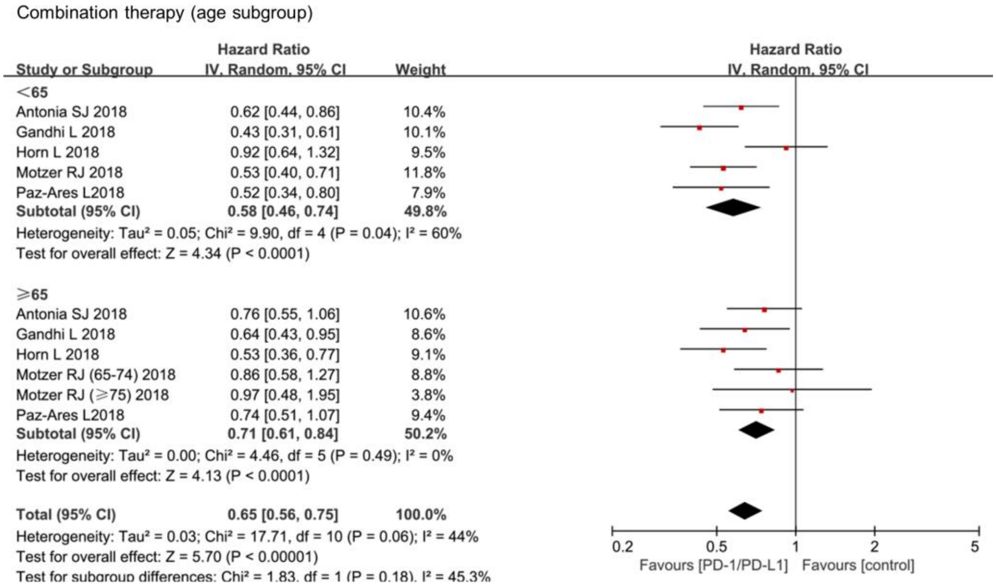

Figure S3 Forest plot of hazard ratio comparing overall survival in patients who received PD-1/PD-L1 inhibitors versus control by age for (A) monotherapy group and (B) combination therapy group.

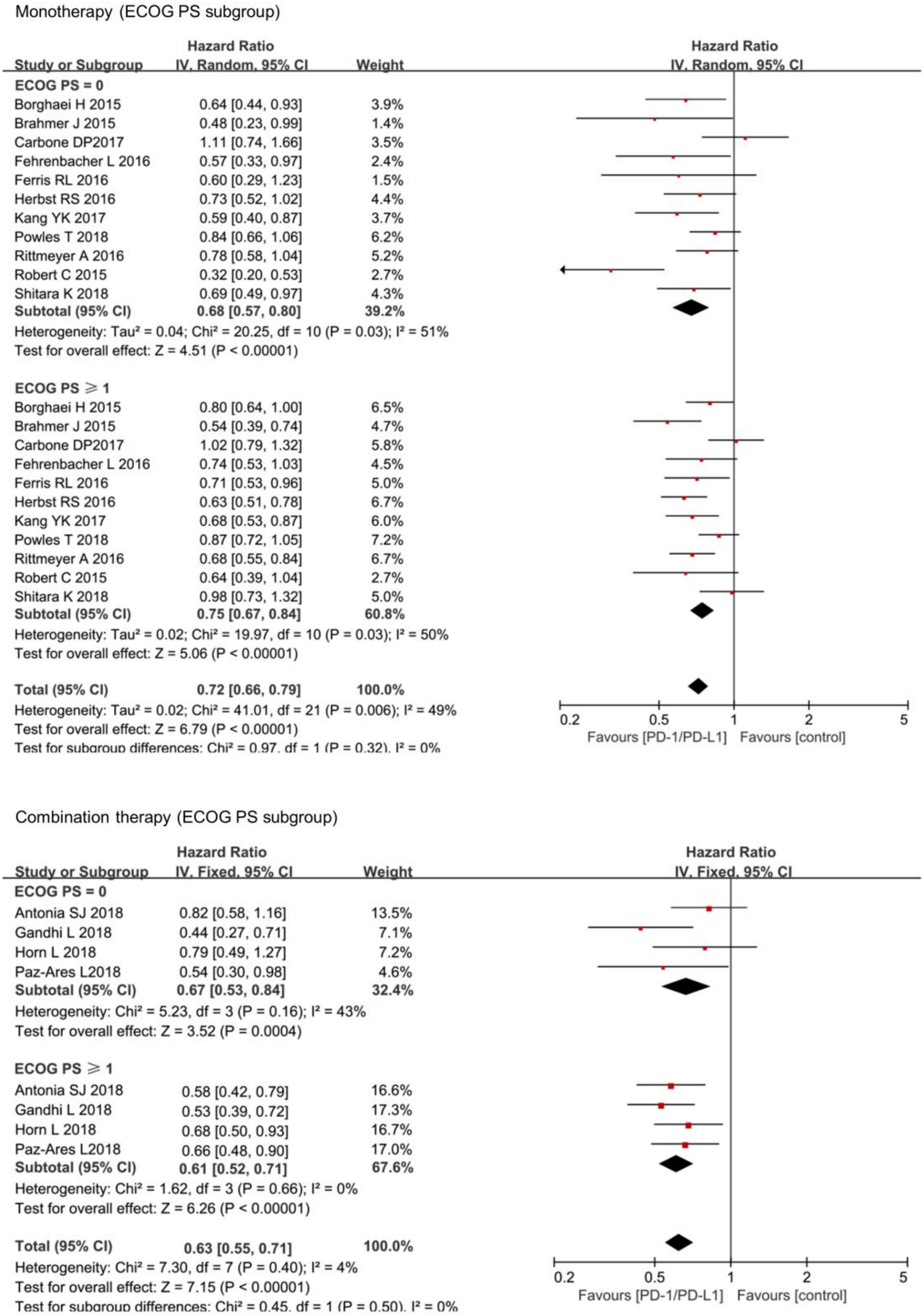

Figure S4 Forest plot of hazard ratio comparing overall survival in patients who received PD-1/PD-L1 inhibitors versus control by ECOG PS for (A) monotherapy group and (B) combination therapy group.

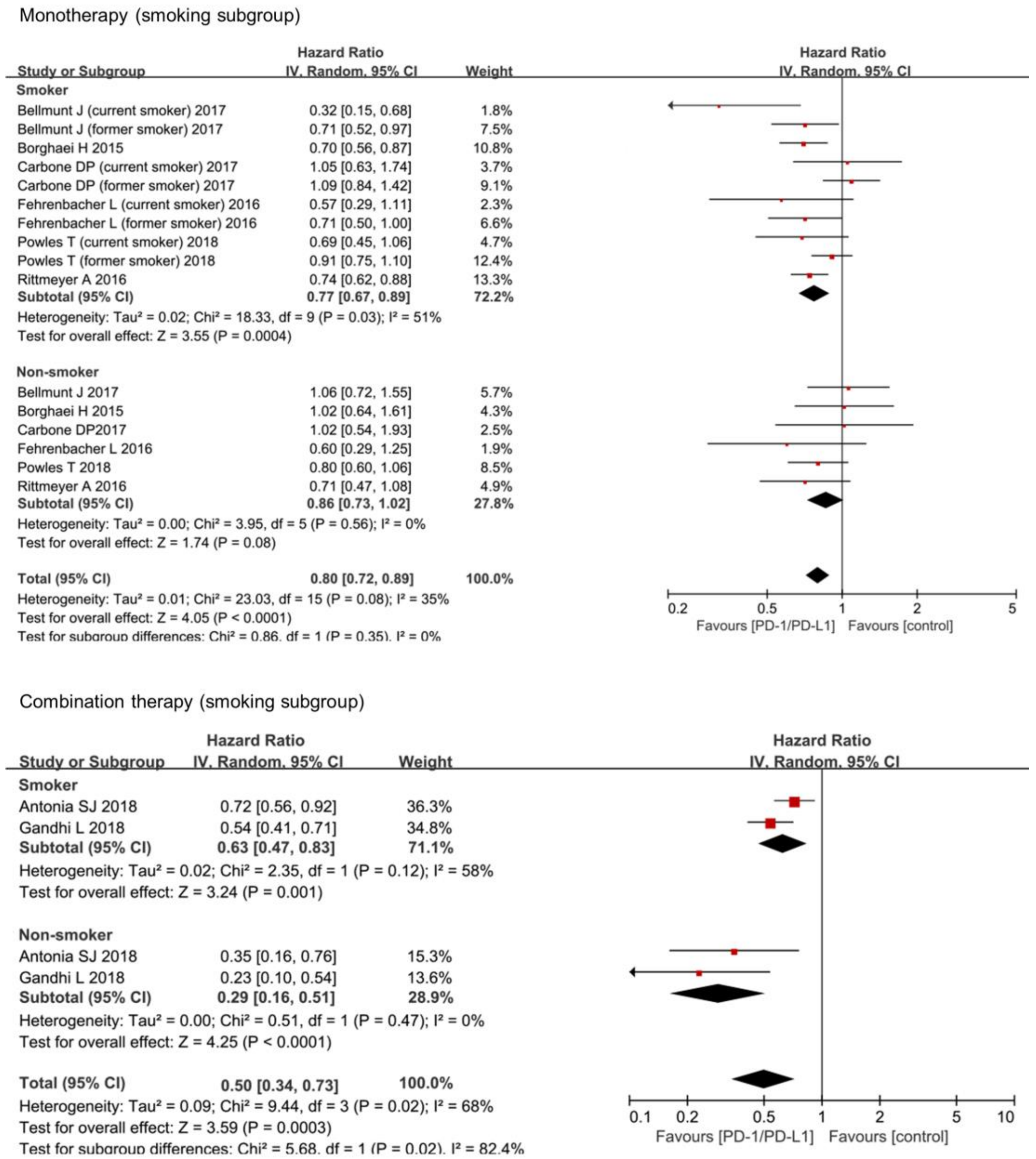

Figure S5 Forest plot of hazard ratio comparing overall survival in patients who received PD-1/PD-L1 inhibitors versus control by smoking status. (A) monotherapy group and (B) combination therapy group.

Monotherapy (liver metastasis subgroup)

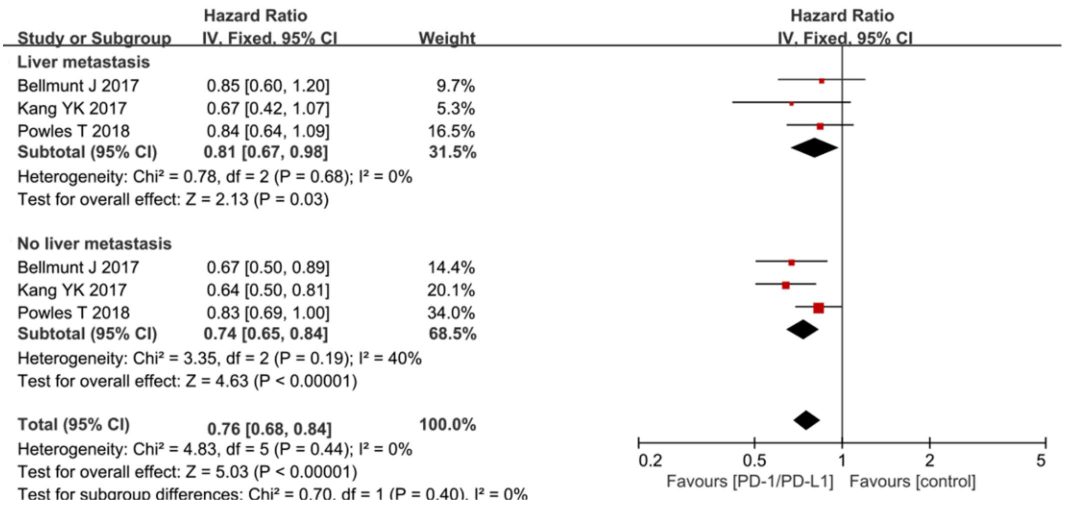

Combination therapy (liver metastasis subgroup)

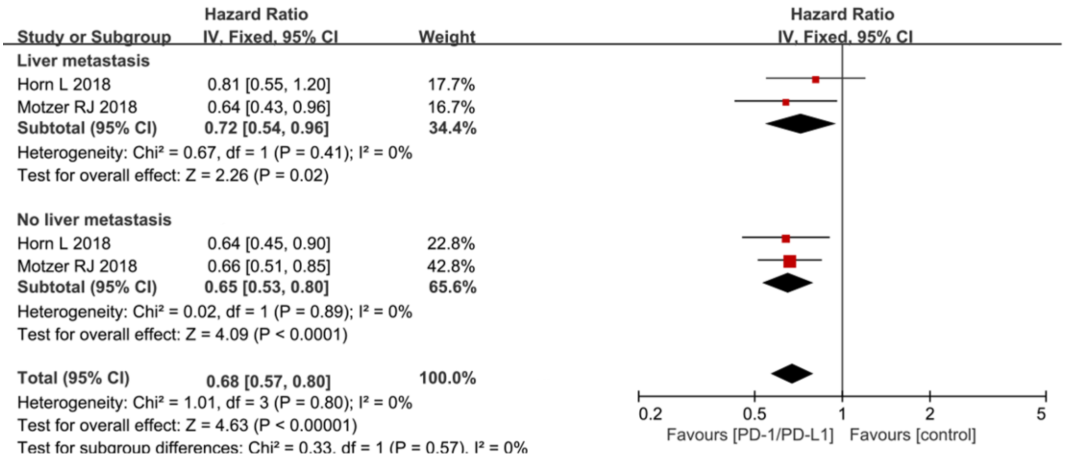

Figure S6 Forest plot of hazard ratio comparing overall survival in patients who received PD-1/PD-L1 inhibitors versus control by liver metastasis for (A) monotherapy group and (B) combination therapy group.

Monotherapy (PD-L1 subgroup)

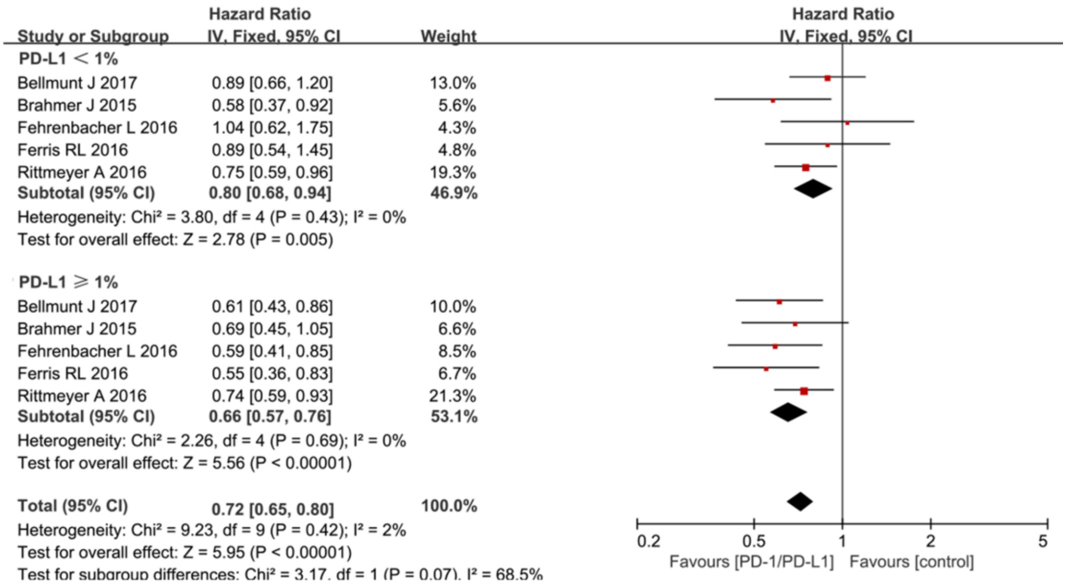

Combination therapy (PD-L1 subgroup)

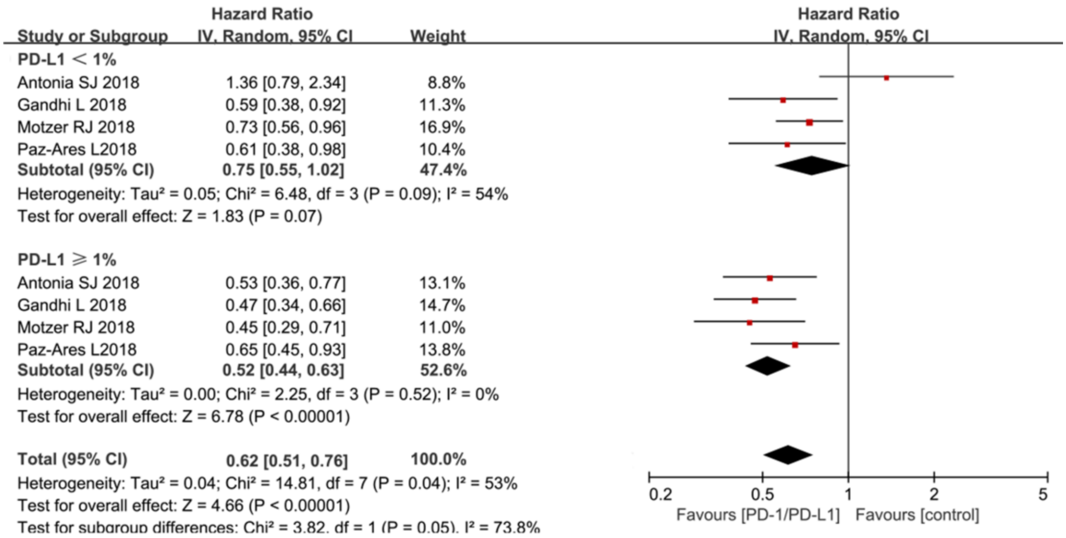

Figure S7 Forest plot of hazard ratio comparing overall survival in patients who received PD-1/PD-L1 inhibitors versus control by PD-L1 expression for (A) monotherapy group and (B) combination therapy group.

Monotherapy (EGFR subgroup)

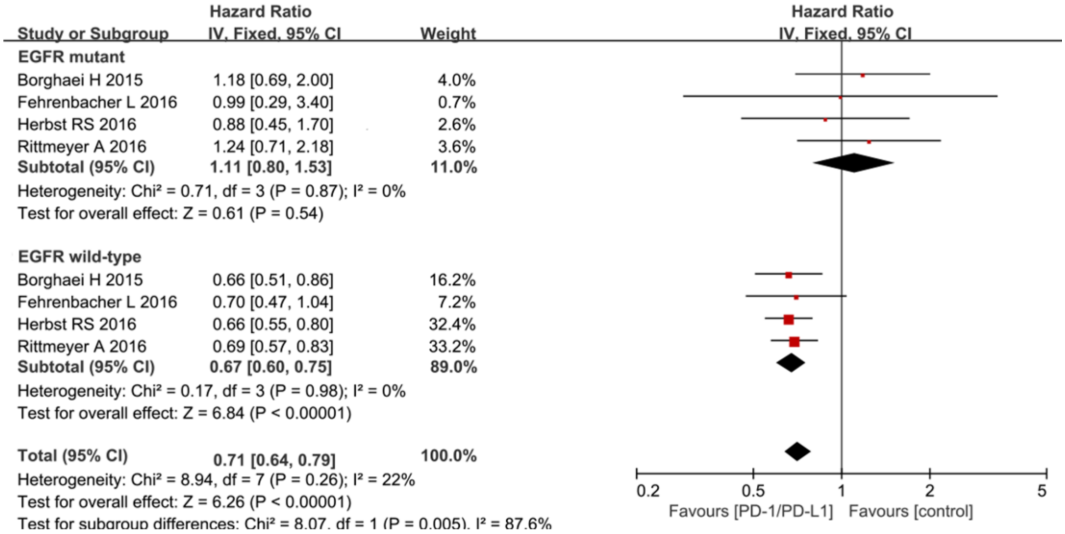

Monotherapy (KRAS subgroup)

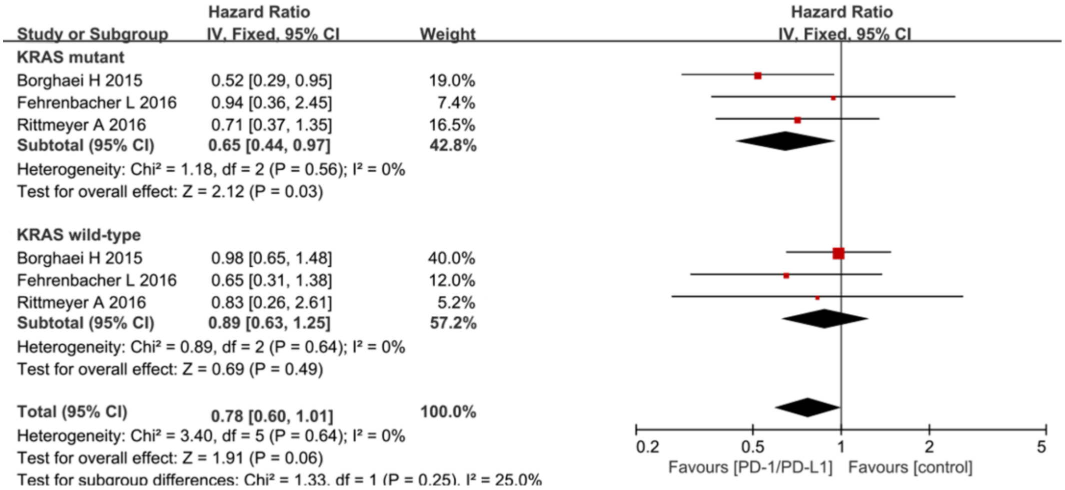

Figure S8 Forest plot of hazard ratio comparing overall survival in patients who received PD-1/PD-L1 inhibitors versus control for monotherapy group by (A) EGFR status and (B) KRAS status.
